# *Salmonella enterica* serovar Typhimurium ST34 co-expressing *bla*_NDM-5_ and *bla*_CTX-M-55_ isolated in China

**DOI:** 10.1038/emi.2017.48

**Published:** 2017-07-12

**Authors:** Lang Yang, Xiaofeng Hu, Xuebin Xu, Chaojie Yang, Jing Xie, Rongzhang Hao, Xinying Du, Ligui Wang, Leili Jia, Peng Li, Shaofu Qiu, Hongbin Song

**Affiliations:** 1 Institute of Disease Control and Prevention, Academy of Military Medical Sciences, Beijing 100071, China; 2 Shanghai Center for Disease Control and Prevention, Shanghai 200236, China

**Dear Editor,**

The *bla*_NDM-5_ gene, which encodes New Delhi metallo-β-lactamase-5, was first reported in 2011 in *Escherichia coli* from a patient in the United Kingdom.^1^ NDM-5 has been reported in many other countries. A variety of plasmid types have been reported to contribute to the widespread dissemination of β-lactam resistance genes. These NDM carriers are mainly identified in *Enterobacteriaceae*, frequently in *Klebsiella pneumoniae* and *E. coli*. Although NDM-1 has been found in *Salmonella enterica*, to our knowledge, no other variants of NDM have been identified in *S. enterica*. Here we characterize the first identification of *Salmonella enterica* serovar Typhimurium (*S.* Typhimurium) strain SSH006 carrying *bla*_NDM-5,_ through routine surveillance in China. This is the first report of the *bla*_NDM-5_-harboring *S.* Typhimurium.

Strain SSH006 was recovered from a fecal sample of a 71-year-old male patient in September 2015 in the enteric clinic of the Minhang district, Shanghai, China. The strain SSH006 was identified as *S.* Typhimurium by amplification and sequencing of the *16S rRNA* gene. The serotype was also determined by using slide agglutination with commercial antiserum (S&A Reagents Laboratory, Bangkok, Thailand) according to the Kauffmann–White scheme. The presence of *bla*_NDM-5_ was determined by PCR screening and was confirmed by sequencing. Southern blotting showed that SSH006 contained two different plasmids (~45 and ~110 kb). The *bla*_NDM-5_ gene was located on the small plasmid, designated pNDM5-SSH006.

Antimicrobial susceptibility testing was performed with a Vitek 2 compact system, and the results were interpreted on the basis of the CLSI guidelines.^2^ The results showed that strain SSH006 was resistant to most tested antibiotics, including imipenem, but was susceptible to aztreonam, amikacin, ciprofloxacin, levofloxacin and nitrofurantoin (Supplementary Table S1). Transferability of the *bla*_NDM-5_ gene was assessed by conjugation experiments using the sodium azide-resistant *E. coli* strain J53 as the recipient. The transconjugants were selected on MacConkey agar plates with antimicrobial drugs. Antimicrobial susceptibility testing revealed that the transconjugants acquired resistance to imipenem, ampicillin and amoxicillin/clavulanic acid (Supplementary Table S1). Unexpectedly, resistance to cephalosporin antibiotics was also observed. Subsequent sequencing revealed that the cephalosporin resistance gene *bla*_CTX-M-55_ was located on the large plasmid, which was simultaneously transferred to the transconjugants. The presence of the *bla*_NDM-5_ and *bla*_CTX-M-55_ genes in the transconjugants was demonstrated by PCR amplification.

The whole genome of *S.* Typhimurium SSH006 was sequenced by the Novogene Company (Beijing, China) on the HiSeq 2500 platform. Paired-end reads of 350 bp were assembled using SOAPdenovo (v2.04) with 109-fold coverage. Multi-locus sequence typing analysis showed that SSH006 belongs to sequence-type 34 (ST34). In addition to *bla*_NDM-5_, multiple resistance genes were identified, including *bla*_TEM-1_, *bla*_CTX-M-55_, *aadA1*, *sul1*, *sul2*, *sul3*, *aac(6’)-Iaa*, *tet(A)*, *floR* and *dfrA12*. Interestingly, there were two *bla*_TEM-1_ genes: one located on the chromosome and the other truncated and located adjacent to the *bla*_CTX-M-55_ gene. A BLAST search indicated that the *bla*_CTX-M-55_-carrying contig covered 5 kb and consisted of a novel combination of *K. pneumoniae* plasmid pKP09085^3^ and *Shewanella sp.* ANA-3 plasmid 1 (CP000470, unpublished).

The complete sequence of the *bla*_NDM-5_-carrying plasmid pNDM5-SSH006 is 46 253 bp in length and shares >99% identity with the IncX3 plasmid pNDM_MGR194 that was isolated in India,^4^ with 17 nucleotide changes. Twelve of the 17 nucleotide changes are located within the truncated *ctuA1* gene, and one is located in the insertion sequence IS*26* downstream of the *bla*_NDM-5_ gene. In addition to these 13 nucleotide variations, the genetic context of *bla*_NDM-5_ in the two plasmids is identical (IS*Swil*-IS*3000*-ΔIS*Aba125*-IS*5*-*bla*_NDM-5_-*ble*-*trpF*-*tat*-Δ*ctuA1*-IS*26*-Δ*umuD*). The shotgun whole-genome sequence and complete sequence of plasmid pNDM5-SSH006 have been deposited in GenBank under accession number MTKV00000000.

A variety of *bla*_NDM-5_-harboring plasmids have been identified and found to share similar sequences with plasmid pNDM_MGR194, such as pEc1929,^5^ pNDM5_0215,^6^ pECNDM101,^7^ and pNDM5-IncX3,^8^ pNDM-QD28 and pNDM-QD29.^9^ Most of these have been isolated in China (Supplementary Table S2). All of the above plasmids had the same genetic context of NDM except pNDM5_0215, which had an insertion of IS*5* within the truncated insertion sequence IS*3000* (Figure 1). Notably, the *bla*_NDM-5_-carrying IncN plasmid pTK1044 from Japan also shared a similar NDM genetic environment with a deletion of the truncated IS*Aba125* but was ~110 kb in length,^10^ thus suggesting a potential dissemination of NDM-5 via mobile genetic elements. Plasmids pJEG027 harboring *bla*_NDM-4_^11^ and pKpN01-NDM7 harboring *bla*_NDM-7_^12^ were also found to be almost identical to pNDM5-SSH006 except for the variation of the *NDM* gene. The *bla*_NDM-7_-harboring IncX3 plasmid pOM26-1 (KP776609, unpublished) is similar to pNDM5-SSH006, except that it has a deletion of IS*Aba125*.

Given that *bla*_NDM-5_ and *bla*_NDM-7_ differ from *bla*_NDM-4_ by a single nucleotide change (G388A and G262T, respectively), the documentation of travel to India indicated that the *bla*_NDM-5_-harboring pNDM_MGR194 and the *bla*_NDM-7_ pKpN01-NDM7 plasmid may have evolved from the *bla*_NDM-4_-harboring pJEG027 plasmid,^12^ thus raising particular concern regarding their epidemic potential to mediate rapid spread of NDM. However, the pNDM5-SSH006-like plasmids were observed in diverse species at different geographical locations across China, in the absence of documented travel history or epidemiological linkage. The increased occurrence of this highly similar IncX3 plasmid indicated the existence of a natural reservoir and rapid transmission of the plasmid in China.

To the best of our knowledge, this is the first report of *S.* Typhimurium carrying the *bla*_NDM-5_ gene. The *bla*_NDM-5_-carrying plasmids have been reported in a narrow host range in *E. coli*, *K. pneumoniae* and recently *P. mirabilis*.^13^ Our work further expands the host range and provides additional evidence of the rapid dissemination of *bla*_NDM-5_ among different species of *Enterobacteriaceae* in China. Given that NDM-5 differs from NDM-1 by two amino-acid substitutions and confers increased resistance to expanded-spectrum cephalosporins and carbapenems,^1^ the ability of this *bla*_NDM-5_-harboring plasmid to transfer across species boundaries may pose a great threat to humans and is quite worrisome. *S.* Typhimurium ST34 clones have raised international concern regarding its rapid spread and multi-drug resistance. A previous study has revealed that *S.* Typhimurium ST34 clones experienced a rapid expansion in China and exhibit a low percentage susceptibility to cephalosporin antibiotics.^14^ However, *S.* Typhimurium ST34 SSH006 exhibited higher resistance to all tested cephalosporin antibiotics.

Here we report the first case of *S.* Typhimurium ST34 SSH006 harboring the *bla*_NDM-5_ gene. The co-existence of two transferable plasmids carrying *bla*_CTX-M-55_ and *bla*_NDM-5_ in *S.* Typhimurium highlights the urgent need for more extensive surveillance and effective action to control its further dissemination.

## Figures and Tables

**Figure 1:**
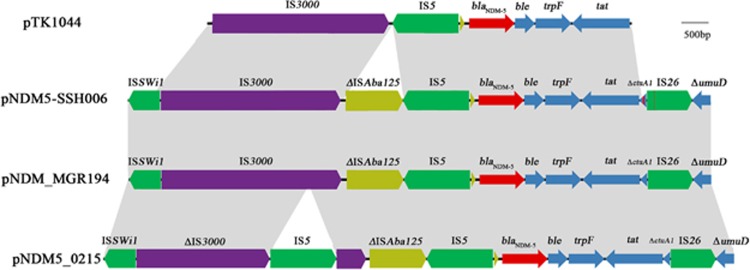
Features of the genetic structure of pTK1044, pNDM5-SSH006, pNDM_MGR194 and pNDM5_0215. A 10.5-kb sequence of the genetic context of the NDM-5-harboring plasmid pNDM5-SSH006 is shown. The arrows indicate open reading frames. Light gray shading denotes homology regions. The *bla*_NDM-5_ gene is shown in red. IS*3000*, IS*Aba125* and the other insertion sequences are shown in purple, yellow and green, respectively. The left genes are shown in blue. Nucleotide changes between pNDM5-SSH006 and pNDM_MGR194 are indicated by red lines.
