# Supplementary material for: Salmonella enterica serovar Typhimurium ST34 co-expressing blaNDM-5 and blaCTX-M-55 isolated in China
Source: Emerg Microbes Infect. 2017 Jul 12;6(7):e61–. doi: 10.1038/emi.2017.48 (PMC5641576; doi:10.1038/emi.2017.48)
Supplement: Supplementary Information [file emi201748x1.doc]

**Supplementary Table S1**. Antibiotic susceptibilities of strain SSH006 and the *E. coli* J53 transconjugants

| Antimicrobial | MIC (μg/ml) | |
| --- | --- | --- |
|  | SSH006 | J53 (the transconjucant) |
| Ampicillin | ≥32 | ≥32 |
| Amoxicillin/clavulanic acid | ≥32 | ≥32 |
| Piperacillin | ≥128 | 64 |
| Cefazolin | ≥64 | ≥64 |
| Ceftazidime | ≥64 | ≥64 |
| Ceftriaxone | ≥64 | ≥64 |
| Cefepime | ≥64 | 8 |
| Aztreonam | ≤1 | ≤1 |
| Imipenem | ≥16 | ≥16 |
| Meropenem | 8 | 2 |
| Amikacin | ≤2 | ≤2 |
| Gentamicin | ≥16 | ≤1 |
| Ciprofloxacin | 0.5 | ≤0.25 |
| Levofloxacin | 1 | ≤0.25 |
| Tetracycline | ≥16 | ≤1 |
| Nitrofurantoin | 32 | ≤16 |
| Sulfamethoxazole/trimethoprim | ≥320 | ≤20 |

***Supplementary Table S2****. bla_NDM_-harboring plasmids Identical to pNDM5-SSH006 in* Enterobacteriaceae

| **Plasmid** | **Species** | **Date** | **Location** | **NDM Type** | **Accession No.** | **Reference** |
| --- | --- | --- | --- | --- | --- | --- |
| pNDM_MGR194 | *K. pneumoniae* | 2011.11-2013.04 | India | *bla*_NDM-5_ | KF220756 | 4 |
| pNDM-QD28 | *E. coli* | 2013.9 | Shandong, China | *bla*_NDM-5_ | KU167608 | 9 |
| pNDM-QD29 | *E. coli* | 2013.9 | Shandong, China | *bla*_NDM-5_ | KU167609 | 9 |
| pEc1929 | *E. coli* | 2014.1 | Hebei, China | *bla*_NDM-5_ | KT824791 | 5 |
| pECNDM101 | *E. coli* | 2015.1 | Sichuan, China | *bla*_NDM-5_ | KX507346 | 7 |
| pNDM5-IncX3 | *K. pneumoniae* | 2015.9 | China | *bla*_NDM-5_ | KU761328 | 8 |
| pNDM5_0215 | *E. coli* | 2013.9 | China | *bla*_NDM-5_ | - | 6 |
| pJEG027 | *K. pneumoniae* | - | Australia | *bla*_NDM-4_ | KM400601 | 11 |
| pKpN01-NDM7 | *K. pneumoniae* | 2013.6 | Canada | *bla*_NDM-7_ | CP012990 | 12 |
| pOM26-1 | *E. coli* | - | Oman | *bla*_NDM-7_ | KP776609 | Unpublished |
